# B‐Cell Differentiation of Human Hematopoietic Progenitors Is Efficiently Supported by Wharton Jelly‐Derived Mesenchymal Stem Cells

**DOI:** 10.1002/eji.70186

**Published:** 2026-04-04

**Authors:** Louison Collet, Hakim Ouled‐Haddou, Hussein Ghamlouch, Walaa Darwiche, Cathy Gomila, Brigitte Gubler, Loïc Garcon, Delphine Lebon, Jean‐Pierre Marolleau

**Affiliations:** ^1^ Laboratoire HEMATIM UR4666 Université Picardie Jules Verne Amiens France; ^2^ Service De Thérapie Cellulaire Centre Hospitalier Universitaire Amiens France; ^3^ Service D'immunologie Centre Hospitalier Universitaire Amiens France; ^4^ Service D'hématologie Biologique Centre Hospitalier Universitaire Amiens France; ^5^ Service D'hématologie Clinique Centre Hospitalier Universitaire Amiens France

**Keywords:** ex vivo B‐cell differentiation, BM‐HSC differentiation, WJ‐MSC coculture

## Abstract

Mesenchymal stem cells (MSC) represent the main stromal component of the bone marrow (BM) niche and are crucial to maintain hematopoietic tissue homeostasis. MSC exhibits extraordinary and multiple properties. In terms of expanding potential and differentiation capacity, Wharton jelly MSC (WJ‐MSC), derived from the umbilical cord, was described as being greater and more performing than MSC from BM or other sources. WJ‐MSC mimics the hematopoietic niche and supports hematopoietic stem cells (HSC) expansion ex vivo. This study aimed to evaluate the effects of human WJ‐MSC cocultured with HSC in a B‐cell differentiation protocol. Remarkably, results highlight WJ‐MSC use as a preferable feeder layer to efficiently support HSC commitment toward the B‐lineage. Over 11 days of HSC coculture with WJ‐MSC, B‐cell genes (*E2A*, *RAG1*, *RAG2*, etc.) expression patterns and B‐cell markers (CD19, immunoglobulin chain, etc.) acquisition were evidenced. WJ‐MSc were also able to unlock the B‐lineage differentiation blockade of the acute lymphoblastic leukemia cell line Nalm16. This model might provide a new strategy to support ex vivo B‐cell differentiation using the powerful properties of WJ‐MSC. This study implements a new approach to improve understanding of B‐leukemogenesis and B‐cell acute lymphoblastic leukemia (B‐ALL) pathophysiology.

## Graphical abstract

Wharton's jelly‐derived mesenchymal stromal cells isolated from umbilical cord were used to recreate a human stromal environment for B‐cell development in vitro. In co‐culture, CD34^+^ hematopoietic progenitors from bone marrow or cord blood differentiated into mature IgM‐expressing B cells by day 11, while the Nalm‐16 B‐ALL cell line showed rapid IgM expression as early as day 5. This model highlights the ability of WJ‐MSC to efficiently support human B‐cell differentiation and maturation in vitro.

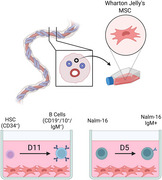

## 1 Introduction

B‐lymphocytes, identified more than half a century ago, are major actors in human health, playing a crucial role in immune defense. They express an antigen receptor (BCR) constituted of a surface immunoglobulin (Ig). The broad diversity of the B‐cell repertoire is generated by V(D)J segments rearrangements of Ig genes, each B‐cell expressing a single specificity. B‐cell development begins with hematopoietic stem cells (HSC) commitment in the hematopoietic niche of the bone marrow (BM), and an ordered process of differentiation steps results in mature B lymphocytes. Interactions with the environment, notably mesenchymal stromal cells (MSC) and cytokine signals, are essential for B‐lineage maturation [1].

Acute lymphoblastic leukemia (ALL) comprises a heterogeneous group of hematologic malignancies characterized by a clonal expansion of immature lymphoid cells [2]. A significant portion of ALL involves the B‐cell lineage, with subsets blocked at different stages of maturation that have challenged optimal treatment development and disease management [2]. Despite progress in this field [3], the mechanisms by which disrupted B‐cell differentiation drives B‐ALL remain incompletely understood. Advances in molecular biology have identified recurrent genetic alterations, such as ETV6::RUNX1, TCF3::PBX1, and BCR::ABL1, as common in B‐ALL [4, 5, 6]. Moreover, Pax5, EBF1, and E2A play essential roles in B‐cell lineage commitment [7]; their loss impairs differentiation, with PAX5 loss even redirecting B‐cells toward alternative fates or apoptosis [8, 9, 10]. Most notably, genes encoding transcriptional regulators of the B‐lineage are mutated in over two‐thirds of B‐ALL and have often been reported to contribute to malignant transformation [9, 10, 11]. Fine mapping of genetic alterations in B‐ALL by contemporary technologies has been tremendously informative, and therapies targeting these genetic abnormalities could allow for more specific treatments liable to improve survival rates and lessen toxicities [12].

Experimental models that faithfully recapitulate dysregulated transcription factor networks in B‐ALL are crucial to gain an understanding of leukemogenesis and drug responsiveness [7]. Murine models are the mainstay of such in vivo immunological experimentations, with a remarkable biological mirror of the human immune system. However, despite this conservation of function, reflected in different reports [13], human and murine B lymphopoiesis exhibit significant differences in immune system development and activation. This can be explained by the diverging evolution of both species since 65 million years ago. To bridge this gap for a better understanding of human B‐cell differentiation, in vitro stromal‐free and stromal‐based models have stimulated interest [14, 15, 16, 17]. Many investigations have highlighted the impact of mesenchymal stromal cells (MSC), which mimic the hematopoietic niche. Notably, the perinatal source of MSC isolated from Wharton jelly (WJ‐MSC) was shown to be an ideal candidate [18]. WJ‐MSC produce several cytokines involved in the regulation of hematopoiesis, in a similar way as that observed in adult BM‐MSC, and express BM‐MSC markers. WJ‐MSC can be easily harvested and usually expand with a high proliferation rate [19]. The relevant potential of WJ‐MSCs for ex vivo expansion of hematopoietic progenitors by direct contact has been reported [20, 21]. Furthermore, Li et al. [22] have reinforced this use of WJ‐MSC and described them as a promising tool for unraveling hematopoiesis physiology.

The role of WJ‐MSC to support B lymphopoiesis in vitro is still unknown, although they have been shown to express a unique secretome and interaction proteins [17, 18]. Hence, it could be interesting to investigate the potential role of WJ‐MSC in the commitment of hematopoietic progenitors toward the B‐cell lineage. WJ‐MSC could represent a new co‐culture model in order to investigate interactions between the early B‐cell differentiation stage of physiologic or leukemic cells with their mesenchymental environment.

The work presented here aimed to establish an ex vivo model using WJ‐MSC to study B‐cell differentiation in both physiological and pathological contexts, focusing on B‐ALL. Through immunophenotyping and gene expression analyses, this unique landscape was explored. Such a well‐defined tool could prove useful to explore potential therapies targeted at specific differentiation stages.

## 2 Materials and Methods

### 2.1 Cell Lines

Nalm16 and KG‐1 cell lines (DSMZ [Deutsche Sammlung von Mikroorganismen und Zellkulturen], Braunschweig, Germany) were cultured in RPMI 1640 medium (Corning, New York, NY, USA) supplemented with 2 mM L‐Glutamine (PAN Biotech GmbH, Aidenbach, Germany), 10 U/mL penicillin‐streptomycin (PS) (Sigma Aldrich, St Louis, MO, USA), and 10% fetal calf serum (FCS, PAN Biotech GmbH). Cells were seeded at a density of 1 × 10^6^/mL for Nalm‐16 and 5 × 10^5^/mL for KG‐1. Medium was refreshed twice a week, and mycoplasma contamination was assessed.

### 2.2 Isolation and Culture of Mesenchymal Cells

WJ‐MSC were obtained from umbilical cords collected at childbirth from consenting mothers. A 1 to 2 cm segment of the umbilical cord was cut out, and Wharton's jelly was collected from 3 mm diameter punches. The latter were seeded in six‐well plates (three punches per well) containing minimum essential medium alpha modification (α‐MEM, Corning) supplemented with 2 mM L‐Glutamine, 10 U/mL PS, and 10% FCS.

BM‐MSCs were obtained from BM samples collected from the Cell Therapy Department of Amiens Picardie University Hospital, with patient consent. Samples were seeded into a T175 flask in proliferation medium α‐MEM supplemented with 2 mM l‐glutamine, 10 U/mL PS, and 10% FCS, and 2 ng/mL β‐fibroblast growth factor (β‐FGF, Sigma Aldrich). After 1 week, the medium was refreshed.

After 2 weeks of culture, adherent cells from both types of samples were harvested by enzymatic dissociation (Trypsin, Sigma‐Aldrich) and reseeded at a density of 3 × 10^4^ cells/well in six‐well plates and 1 × 10^6^ cells in T75 flasks.

### 2.3 Functional Characterization of MSC

For functional characterization, MSCs were seeded at a density of 2 × 10^4^ cells/well in six‐well plates and cultured for 21 days in the presence of proliferation medium for MSCs. The differentiation medium consisted in Dulbecco's Modified Eagle Medium (DMEM‐low glucose, Sigma Aldrich) supplemented with 10% FCS (Eurobio, Les Ulis, France), 2 mM L‐glutamine (Sigma Aldrich), 10 U/mL penicillin‐streptomycin antibiotic solution (Sigma Aldrich), 0.5 mM IBMX (Isobutylmethyl xanthine, Sigma Aldrich), 60 µM indomethacin (Sigma Aldrich), and 1 nM dexamethasone (Sigma Aldrich). Lipid vacuoles, which are adipocytic differentiation markers, were identified by RedOil staining (Sigma Aldrich) according to manufacturer instructions. Images were captured at x4, x10, and x20 magnification using a Nikon Eclipse TS100 inverted microscope equipped with a Nikon 1v2 camera (Nikon, Tokyo, Japan).

### 2.4 Co‐Culture Model Setup

HSC were isolated from leukapheresis or density gradient centrifugation of bone marrow samples. CD34^+^ cells were selected by magnetic‐activated cell sorting (MACS) using the CD34 MicroBeads kit (Miltenyi Biotec, Bergisch Gladbach, Germany) according to manufacturer recommendations on an autoMACS pro separator (Miltenyi Biotec).

Once enumerated, sorted CD34^+^ cells or cell lines (Nalm16, KG‐1) were seeded in direct co‐culture in six‐well plates, precoated with 3 × 10^4^ feeder cells (WJ‐MSCs, BM‐MSCs, or MS‐5).

B‐cell differentiation medium consisted of Iscove's Modified Dulbecco's Medium (IMDM, Biochrome, Cambridge, UK) supplemented with 2 mM L‐Glutamine (Sigma Aldrich), 10 U/mL penicillin‐streptomycin antibiotic solution (Sigma Aldrich), 100 ng/mL stem cell factor (SCF, Miltenyi Biotec), 10 ng/mL FMS‐like receptor tyrosine kinase‐3 ligand (Flt‐3L, Miltenyi Biotec), and 25 ng/mL IL‐7 (IL‐7, Miltenyi Biotec). All experiments were performed using a recombinant, chemically defined human serum alternative (H2B, Dominique Dutscher, Bernolsheim, France) at a low concentration (2.5%). H2B is an artificial protein matrix, free of bovine growth factors, exosomes, and hormone contaminants, and manufactured under controlled conditions ensuring lot‐to‐lot reproducibility. Sequential sampling was performed on days (D) 0, D3, D5, D7, and D11 for immunophenotypic analyses.

WJ‐MSCs were generated from four independent umbilical cord donors (*n* = 4). Functional assays were performed using three independent BM‐HSC samples (*n* = 3), three independent WJ‐MSC donors for PB‐HSC assays, and four independent WJ‐MSC donors for Nalm‐16 assays. In all cases, one replicate corresponds to one CD34+ cell donor co‐cultured with one distinct WJ‐MSC donor. All stromal layers used were derived from WJ‐MSCs expanded to passage 2. To mimic physiological stromal dynamics, WJ‐MSCs were not cell‐cycle arrested before co‐culture. WJ‐MSC feeder layers were refreshed once per week, and the cytokine cocktail (IL‐7, SCF, FLT3L) was replenished twice per week, according to established hematopoietic differentiation protocols.

### 2.5 Immunophenotyping and Cell Sorting

For immunophenotyping studies, cells were harvested, counted, and incubated in phosphate buffer saline (PBS) supplemented with 0.5% BSA and 2 mM EDTA along with fluorophore‐conjugated antibodies according to supplier recommendations. An immunoglobulin constant fraction blocker (FcR block buffer, Miltenyi Biotec) diluted at 1/100 was used to prevent antibody capture by myeloid cells (monocytes and macrophages). The antibody panel is described in Table S1.

B‐cell differentiation stages were defined as follows:

- HSC stage:CD34^+^/CD10^−^/CD19^−^:
- Committed lymphoid progenitor (CLP)/preproB stage: CD34^+^/CD10^+^/CD19^−^:
- proB stage: CD34^+^/CD10^+^/CD19^+^:
- pro‐preB stage: CD34−/CD10+/CD19+/CD20−
- preB stage : CD34^−^/CD10^+^/CD19^+^/CD20^+^/cytoplasmic μ chain+
- late preB/immature B‐cell: CD34^−^/CD10^±^/CD19^+^/CD20^+^/surface μ chain+

After co‐culture of Nalm16 cells with WJ‐MSC, cells were sorted according to the expression or non‐expression of CD43 and μ chains using a BD FACS Melody cell sorter (BD Biosciences, Franklin Lakes, NJ, USA).

### 2.6 Quantitative Real‐Time PCR (qRT‐PCR)

Cells for which nucleic acids had to be extracted were collected, counted, and then frozen at −80°C in RLT lysis buffer (Qiagen, Hilden, Germany). RNAs were extracted using the RNeasy Mini Kit (Qiagen), according to manufacturer instructions. Extracted RNA was then stored at −80°C until use. RNA concentration and purity were verified using a NanoDrop spectrophotometer using 280/260 or 260/230 ratios. Complementary DNA (cDNA) was generated by reverse transcription of 1 µg extracted RNA with the High‐Capacity cDNA Reverse Transcription Kit (ThermoFisher, Waltham, MA) according to manufacturer instructions.

Amplification reactions were carried out on 50 ng of cDNA in a final volume of 20 µL. The mix consisted of Power SYBR Green PCR Master Mix (ThermoFisher) with UNG, and 500 nM each of forward and reverse primers (Table S2). The amplification reaction, including a melting curve step, was performed on a QS7 quantitative thermocycler (Applied Biosystems, Waltham, MA, USA) according to the manufacturer's instructions. All qPCR experiments were performed in triplicate.

### 2.7 Statistical Analysis

An Agostino–Pearson test was conducted to determine whether samples followed a normal distribution. Based on normality test results, different groups were compared pairwise, using *T*‐tests or analysis of variance (ANOVA). Statistical analyses were performed using GraphPad Prism software (Version 10.0.0, GraphPad Software, San Diego, CA, USA). Data are expressed as mean ± standard deviation (SD), and significance was set at *p* < 0.05.

## 3 Results

### 3.1 Immunophenotype and Differentiation Capacity of WJ‐MSCs

WJ‐MSC immunophenotype was first investigated by FCM with an appropriate panel according to the criteria proposed by the International Society for Cellular Therapy [25]. This disclosed the expected expression of CD90, CD105, CD73, CD44, and CD166 mesenchymal markers, in the absence of CD34, CD45, CD14, and HLA‐DR hematopoietic markers. BM‐MSC, used as a positive control for mesenchymal marker expression, confirmed the mesenchymal immunophenotype of WJ‐MSC. Representative results of three independent experiments are shown in Figure S1A.

WJ‐MSC functionality was analyzed by evaluating adipocytic differentiation through the accumulation of lipid vacuoles. Representative images from three independent experiments are shown in Figure S1B.

### 3.2 WJ‐MSC Maintain HSC Expansion and Support B‐cell Differentiation

**FIGURE 1:**
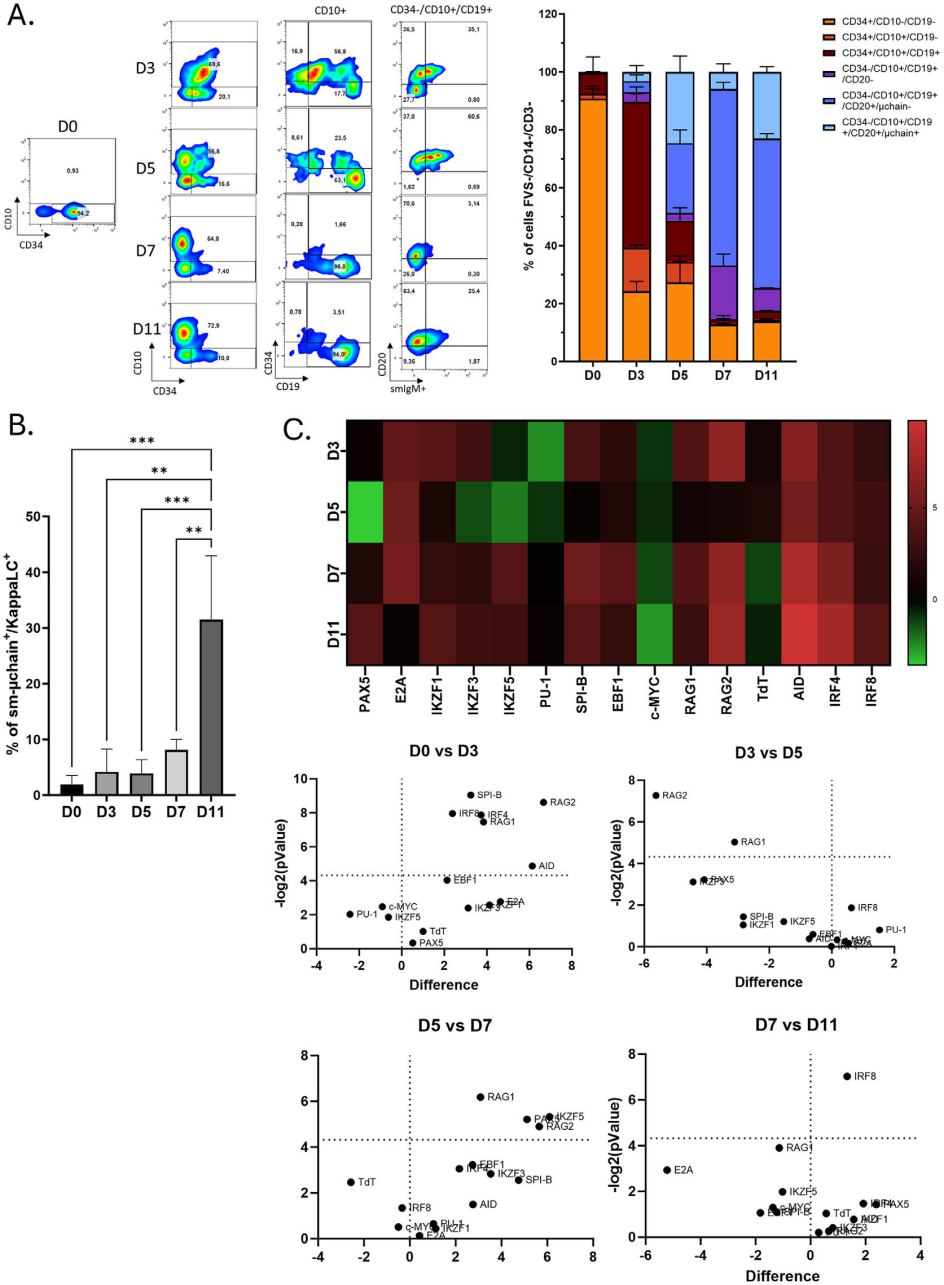
Induction of B‐cell differentiation from BM‐HSC on WJ‐MSCs support: (A) Flow cytometry plots illustrating the gating strategy for B‐cell differentiation in co‐cultures of Wharton's Jelly mesenchymal stromal cells (WJ‐MSCs) with bone marrow‐derived hematopoietic stem cells (BM‐HSCs). Cells were gated on singlets and negative for fixable viability stain (FVS), CD14, and CD3 to exclude dead cells, monocytes, and T‐cells, respectively. Data are representative of three independent experiments (*n* = 3). (B) Histogram showing the percentage of cells expressing surface membrane μ‐chain and κ light chain (sm‐μ‐chain^+^/κappaLC^+^) at different time points of the WJ‐MSC co‐culture (D0, D3, D5, D7, D11). A significant increase in sm‐μ‐chain^+^/κappaLC^+^ cells is observed from day 7 onward, indicating progressive B‐cell differentiation. Data represent mean ± SEM of three independent experiments. Statistical analysis was performed using a one‐way ANOVA test (**p < 0.01, ***p < 0.001). (C) Heatmap representing RT‐PCR expression profiles of key B‐cell transcription factors and lineage‐specific genes, including *PAX5, IKZF1, IKZF3, PU.1, EBF1, E2A, IRF4, IRF8, RAG1, RAG2, TdT*, and *AID*. Gene expression levels are shown relative to undifferentiated control cells, with red indicating upregulation and green, downregulation. Comparisons were made between samples collected at different time points of the co‐culture to assess the temporal dynamics of gene expression. Differential gene expression analysis is further visualized in a volcano plot. The vertical threshold line indicates the fold‐change cutoff for significant upregulation or downregulation, while the horizontal line represents the statistical significance threshold (*p* < 0.05).

During the co‐culture of sorted CD34^+^ BM‐HSC with WJ‐MSC adherent cells, HSC progressively differentiated toward B‐cells (Figure 1A). At D0, undifferentiated CD34^+^/CD10^−^/CD19^−^ cells represented 90.7 ± 4.3% of the population. By D3, 50.4 ± 2.7% of CD34^+^/CD10^+^ cells displayed a pro‐B CD34^+^/CD10^+^/CD19^+^ immunophenotype. On Day5, 48.0 ± 9.9 % of CD34^+^/CD10^+^ cells additionally expressed CD20. Finally, from D7 on, more than 80% of the cells had lost membrane CD34. Differentiation continued up to early B‐cells expressing surface mu chain as early as D5:(25.6 ± 5.5%), and even light chain co‐expression (mature μk sIg B‐cells) on D11 (23.0 ± 1.75%) (Figure 1B).

Gene expression of B‐lineage transcription factors (Figure 1C) showed on D3, a statistically significant overexpression of *SPIB*, *RAG1*, *RAG2*, *IRF4*, *IRF8*, and *AID* compared with D0. *RAG1* and *RAG2*, still detectable on D5, were then repressed. In parallel, *PAX5* and *IKZF5* transcripts became detectable, as well as *IRF8* on D7 and D11.

Comparable results were obtained using peripheral blood hematopoietic stem cells (PB‐HSC) for the co‐culture (Figure S2A). In the absence of stromal support, and despite exposure to the same IL‐7/SCF/FLT3L cocktail, cells remained arrested at the CD34^−^CD10^+^CD19^−^ stage, indicating that cytokines alone are insufficient to trigger the CD10^+^ to CD19^+^ transition (Fig S2B).

### 3.3 B‐Cell Differentiation Is Substantially More Efficient in WJ‐MSC Coculture Than With the Murine Stromal Cell Line (MS‐5)

**FIGURE 2:**
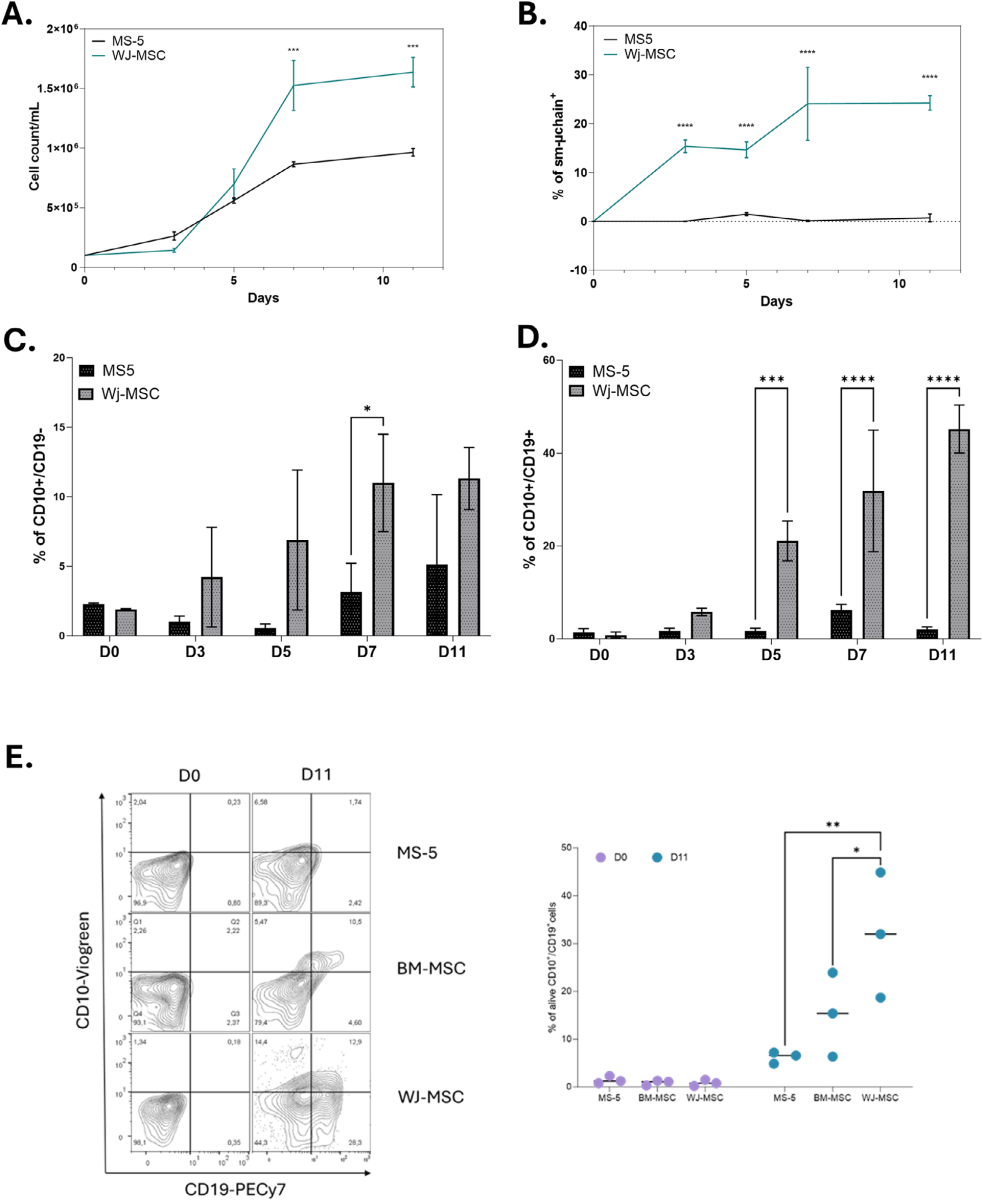
Comparison of B‐cell differentiation potential between MS‐5 and WJ‐MSC co‐cultures: (A) Total cell expansion over time in co‐cultures of BM‐HSCs with either MS‐5 or WJ‐MSC feeder layers. Cell counts were performed at days 0, 3, 5, 7, and 11. (B) Frequency of surface μ‐chain (sm‐μ‐chain^+^) expressing cells over time. (C) Percentage of CD10^+^CD19^−^ early B‐cell precursors at each time point (D). Flow cytometry plots illustrating the gating strategy for B‐cell differentiation in co‐cultures of a comparison between MS‐5, BM‐MSC, and WJ‐MSC (E.) with peripheral blood hematopoietic stem cells (PB‐HSCs). Percentage of CD10^+^CD19^+^ B‐lineage committed cells over time. Data represent mean ± SEM of three independent experiments. Statistical significance was determined using a two‐way ANOVA comparison test. *p < 0.05; ***p < 0.001; ****p < 0.0001.

The characteristics of HSC co‐cultures with WJ‐MSC were compared with those commonly used with the murine stromal cell line MS‐5. Cell proliferation was significantly higher with WJ‐MSC at D7 (1.5 × 10^6^ ± 0.2 × 10^6^ cells vs 0.9 × 10^6^± 0.2 × 10^6^; Figure 2A). The three maturation stages, respectively pre‐B (sμ), early (CD10^±^/CD19^−^), and pro‐B (CD10^+^/CD19^+^), were also present at significantly higher percentages with WJ‐MSC, respectively from D3, D7, and D5 on (Figure 2).

To further evaluate the ability of stromal cells to support B‐cell differentiation, CD34^+^ hematopoietic progenitors were cultured on MS‐5, BM‐MSC, or WJ‐MSC layers and analyzed by flow cytometry at day 11. Representative contour plots are shown in Figure 2E (left). Both BM‐MSC and WJ‐MSC cultures showed a higher proportion of CD10^+^/CD19^+^ cells compared with MS‐5 conditions (MS‐5 vs. BM‐MSC: 6.207 ± 1.214 vs. 15.223 ± 8.766, *p* = 0.0212; MS‐5 vs. WJ‐MSC: 6.207 ± 1.214 vs. 31.867 ± 13.101, *p* = 0.0011). No significant difference was observed between BM‐MSC and WJ‐MSC conditions.

### 3.4 WJ‐MSC Can Unlock B‐Cell Differentiation Blockade in the B‐ALL Cell Line Nalm‐16

**FIGURE 3:**
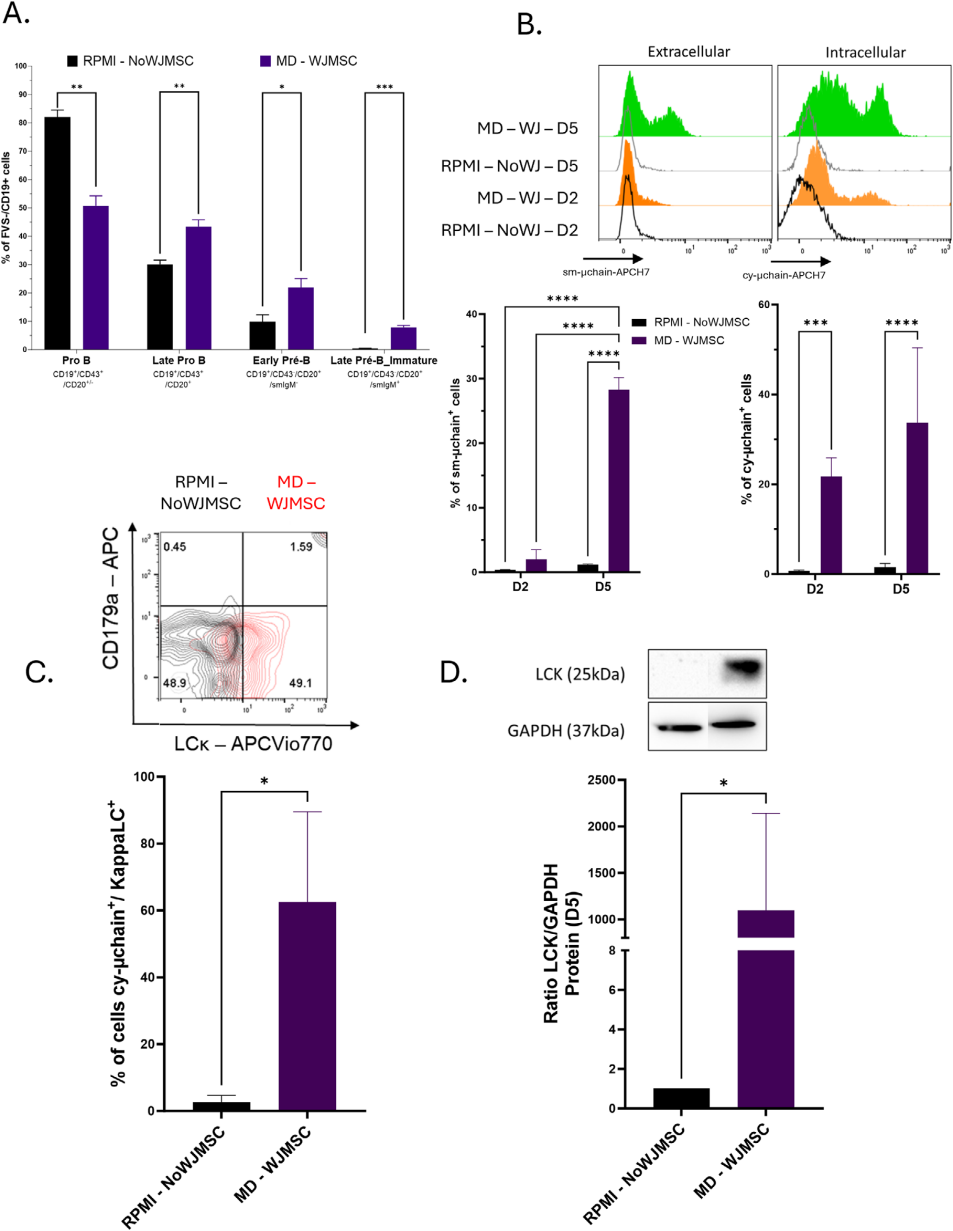
Unblocked B‐cell differentiation and LCK expression in the Nalm‐16 cell line: (A) Flow cytometry analysis of B‐cell developmental subpopulations under noninduced (RPMI—NOWJMSC), black) and induced (MD—WJMSC, purple) conditions. Bar graphs represent the frequency of cells within defined stages of B‐cell development, based on expression of surface markers (indicated below each bar, e.g., CD19, CD10, CD34, CD20, IgM). Data are shown as mean ± SEM from *n* = 3 independent experiments. (B) Flow cytometry analysis of μ‐chain expression at the cell surface (left panels) and intracellularly (right panels) at day 2 and day 5 of differentiation. (C) Expression of CD179a (VpreB) and LCK in NI vs. IND conditions. Representative dot plot and quantification of cy‐μ chain^+^LCK^+^ cells. (D) LCK protein expression was assessed by Western blot in NI and IND cells. Representative blot (upper panel) and densitometric quantification relative to GAPDH (lower panel) are shown (mean ± SEM, *n* = 4). Lanes were cropped for presentation purposes. Full‐length, uncropped blots are provided in Figure S1. Statistical analysis was performed using unpaired *t*‐test, one‐way ANOVA, or two‐way ANOVA. *p < 0.05; **p < 0.01; ***p < 0.001; ****p < 0.0001.

Co‐culture of WJ‐MSC with Nalm16 cells (Figure 3A) demonstrated a progressive maturation of these ALL cells as of D5. Approximately 30% of Nalm16 cells no longer expressed the early stage marker CD43 (29.79% vs. 10.38% for Nalm16 cells alone). Moreover, 8% of co‐cultured Nalm16 cells expressed intracytoplasmic, then surface μ chains (Figure 3B). By D5, kappa light chain could also be detected in FCM, although no surrogate light chain (CD179a VpreB) expression could be evidenced (Figure 3C). Kappa light chain was also detected in the western blot (Figure 3D) at D5. FCM also showed an inverse correlation to the expression of μ chains and the IL‐7 receptor CD127. Beyond day 5, a marked reduction in Nalm‐16 cell viability was observed under our culture conditions, which precluded reliable assessment of later differentiation stages or long‐term proliferative behavior (data not shown).

**FIGURE 4:**
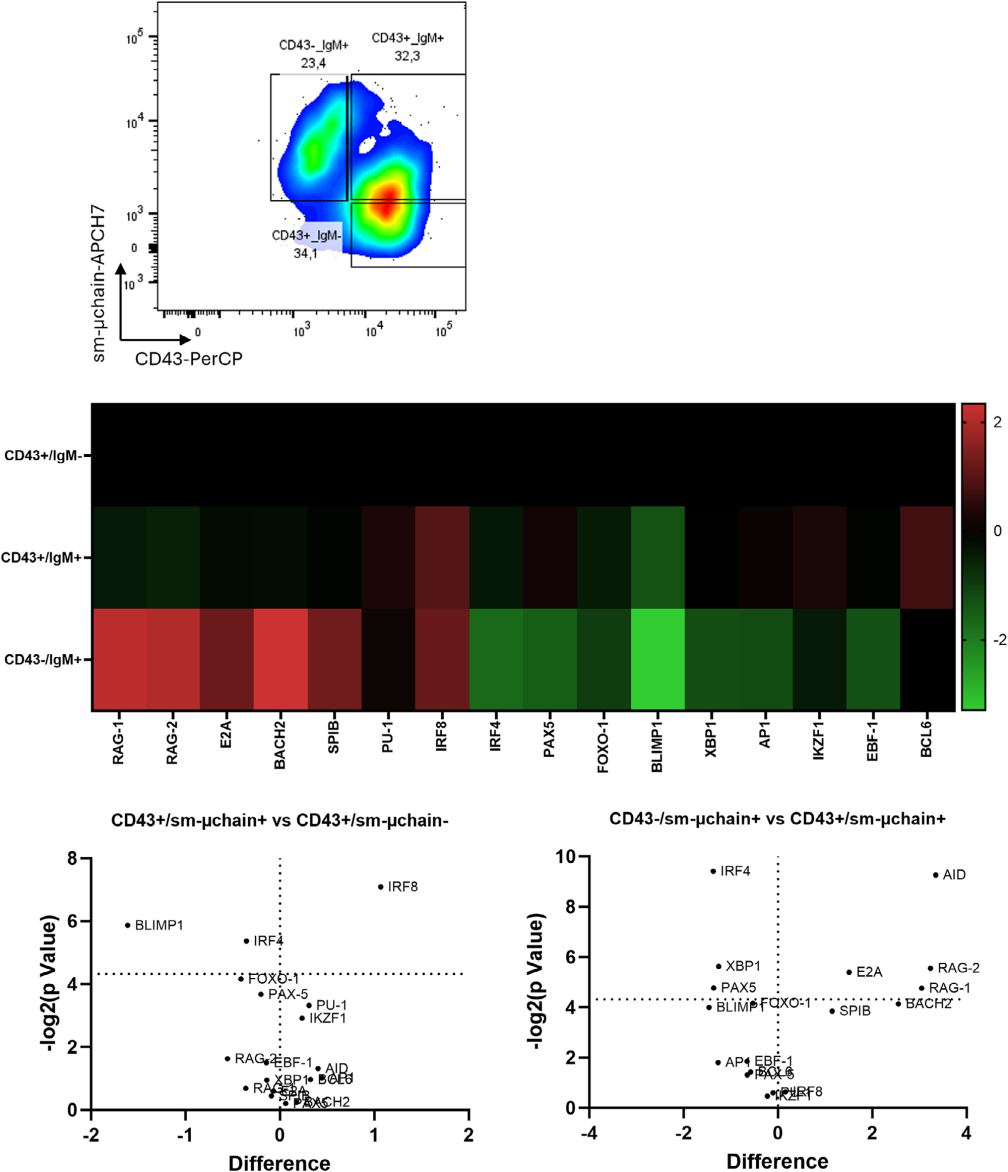
Transcriptional profiling of sorted B‐cell subpopulations based on CD43 and smμ‐chain expression at D5: (A) Gating strategy used for cell sorting using BD FACSMelody. Cell populations were gated based on CD43 and surface μ‐chain (sμ‐chain) expression, allowing isolation of three distinct subpopulations: CD43^−^/sμ‐chain^+^, CD43^+^/sμ‐chain^+^, and CD43^+^/sμ‐chain^−^. (B) Heatmap (top) showing relative mRNA expression of key transcription factors and B‐lineage genes in the three sorted subpopulations. Expression levels are normalized (Z‐score) and color‐coded (green = downregulated, red = upregulated). Volcano plots (bottom) represent differential gene expression between CD43^+^/sμ‐chain^+^ vs. CD43^−^/sμ‐chain^+^ (left), and CD43^+^/sμ‐chain^−^ vs. CD43^+^/sμ‐chain^+^ (right). Selected genes are labeled. Dashed lines indicate thresholds for statistical significance (*p* < 0.05) and fold change.

Analysis of the Nalm16 transcriptomic profile (Figure 4) was performed. Three populations were obtained by cell‐sorting, respectively, CD43^+^/μ^−^, CD43^+^/μ^+^, and CD43^−^/μ^+^ (Figure 4A). Matured Nalm16 displayed a significant downexpression of *BLIMP1*, *PAX5*, and *IRF4*, together with *IRF8* overexpression. The essential Ig‐recombination *RAG‐1*, *RAG‐2*, *AID*, and *E2A* genes were also significantly overexpressed in matured CD43^−^/μ^+^ cells compared with control Nalm16 cells (Figure 4B).

Finally, co‐culture of WJ‐MSC with an acute myeloblastic leukemia cell‐line (KG‐1) was performed as a control of the B‐lineage specificity of WJ‐MSC differentiation capacities. After 5 days of culture, no difference in the CD34^+^/CD33^+^/CD14^−^/CD10^−^/CD19^−^ immunophenotype of KG‐1 cells was observed (Figure S3).

## 4 Discussion

This study sheds light on the intricate interplay between WJ‐MSCs and HSC B‐cell differentiation, providing valuable insights into the supportive role of human WJ‐MSC cells for ex vivo studies investigating B‐cell differentiation pathways.

In a physiological context, WJ‐MSCs were demonstrated to efficiently support BM or PB HSCs differentiation toward the B lineage in a novel co‐culture model. Over 11 days, a tenfold increase in B lymphocytes was observed, through all stages of B‐cell lymphopoiesis. These data are in line with previous reports about in vitro B‐cell maturation [16], yet with a faster schedule. We note that the WJ‐MSC secretome has been previously profiled in depth, confirming that WJ‐MSCs are known to produce high CXCL12 levels and other trophic factors, consistent with their enhanced hematopoietic‐supportive capacity compared with adult BM‐MSCs [26, 27, 28, 29, 30, 31]. The acquisition of CD19 on about 80% of the cells observed here is close to the 72%–80% reported by Kraus et al. in a cell‐free model, as well as the appearance of surface μ/k chains, respectively at 31.5% vs. 21.5%–78.9% [14]. Kraus et al. also showed that cord blood HSCs in their model could reconstitute the B‐cell repertoire in newborn *Rag2^−/−^ IL2rg^−/−^* mice [17]. Future investigations should explore the reconstructive capacity of isolated subpopulations obtained in the WJ‐MSC model reported here.

Murine stromal cell lines such as MS‐5 have been widely used as surrogate niche models [13, 19, 20] for HSC differentiation. WJ‐MSCs offer the advantage of a human origin and appear to better recapitulate the BM microenvironment than MS‐5 to promote B‐cell differentiation from human HSC. The latter already reached the pre‐B stage by D7, a result taking around 6 weeks with MS‐5 protocols, with a lower output of about 12% [32] vs. 20% with WJ‐MSC. This 20% rate is interesting, taking into account the fact that physiological B‐cell differentiation involves immunoglobulin gene rearrangement failures leading to a positive selection of cells with both productive heavy (IgH) and light (IGK or IGL) chain alleles. The WJ‐MSC model was also shown to recapitulate the sequence of B‐lineage gene expression profiles during differentiation. Again, this was previously observed in a model using BM‐MSC [16], yet after 42 days, instead of the 11 days of the WJ‐MSC. The only human feeder‐free model published to date requires 45 days to reach stages [17] that are achieved within 11 days in our WJ‐MSC system. This distinction contextualizes the superior efficiency and relevance of our stromal‐supported approach.

**FIGURE 5:**
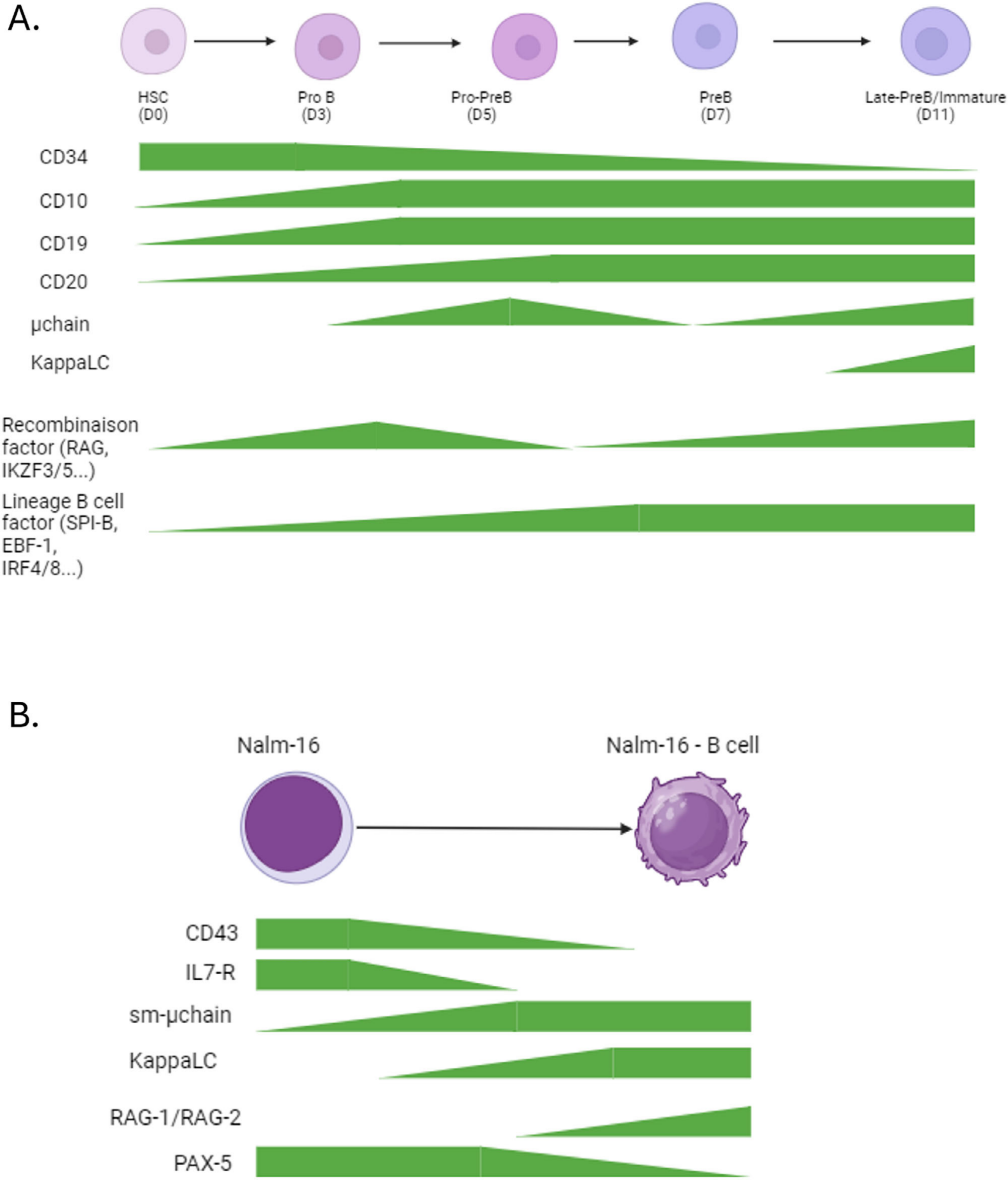
Schematic representation of B‐cell differentiation dynamics and marker expression in the models reported here (BM‐HSC, A; Nalm16, B).

Of note, WJ‐MSC were shown to be efficient for the differentiation of BM‐HSC but also PB‐ HSC. All in all, WJ‐MSC appears to be a great candidate for in vitro B‐cell differentiation protocols. The conventional 2D culture protocol presented is easy to set up and effective in promoting HSC maintenance/expansion/differentiation. But some evidence suggests that a 3D MSC architecture would mimic better physiological conditions. Indeed, as described by Ferreira et al., a 3D fibrin scaffold with MSC has been shown to support cord blood‐HSC expansion efficiently. Recently, Fereydani et al. demonstrated that WJ‐MSC on a 3D coculture system effectively increased HSC expansion and maintained their colonization potential. Thus, the possible conversion of the WJ‐HSC 2D model reported here into a 3D system could improve B differentiation. Moreover, since recent studies have clarified the role of the BM microenvironment in the pathogenesis of hematologic cells, this model could be exploited to identify target molecules promoting leukemogenesis (Figure 5).

## Author Contributions

L.C. designed the study, performed experiments, analyzed the data, and wrote the manuscript. H.O.‐H., H.G., W.D., and C.G. contributed to experiments and data acquisition. B.G. and L.G. provided essential materials and technical expertise. D.L. and J.‐P.M. supervised the study and critically revised the manuscript. All authors contributed to data interpretation and approved the final version of the manuscript.

## Conflicts of Interest

The authors declare no conflicts of interest.

## Supporting information




**Supporting File**: eji70186–sup–0001–SuppMat.pdf.

## Data Availability

The data supporting the findings of this study are available from the corresponding author upon reasonable request.
