# Supplementary material for: B‐Cell Differentiation of Human Hematopoietic Progenitors Is Efficiently Supported by Wharton Jelly‐Derived Mesenchymal Stem Cells
Source: Eur J Immunol. 2026 Apr 4;56(4):e70186. doi: 10.1002/eji.70186 (PMC13049500; doi:10.1002/eji.70186)
Supplement: Supplementary file 1 — Supporting File: eji70186–sup–0001–SuppMat.pdf. [file EJI-56-e70186-s001.pdf]

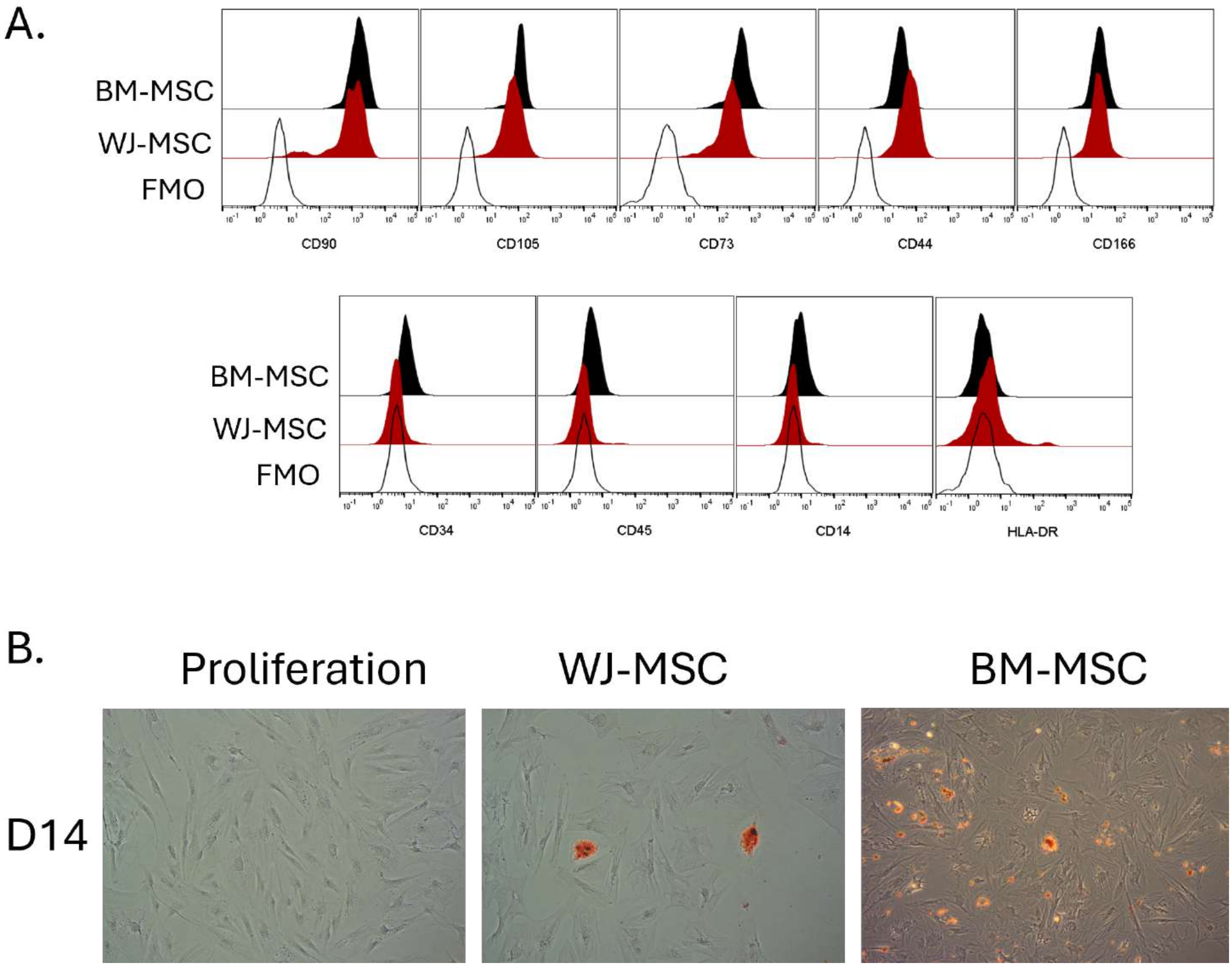
A.
BM-MSC
WJ-MSC
FMO
CD90
CD105
CD73
CD44
CD166
CD34
CD45
CD14
HLA-DR
B.
Proliferation
WJ-MSC
BM-MSC
D14

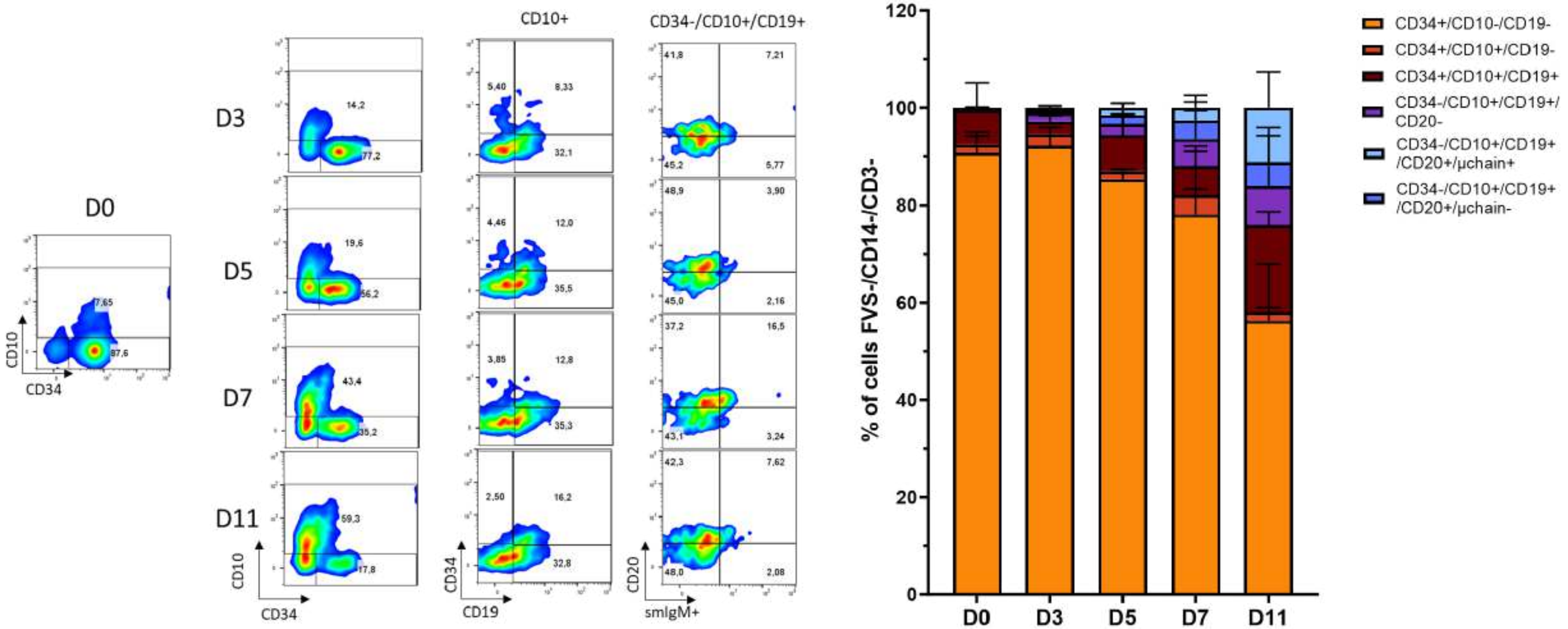
D0
D3
D5
D7
D11
CD10+
CD34-/CD10+/CD19+
CD10
CD34
CD19
CD20
smIgM+
% of cells FVS-/CD14-/CD3-
CD34+/CD10-/CD19-
CD34+/CD10+/CD19-
CD34+/CD10+/CD19+
CD34-/CD10+/CD19+/CD20-
CD34-/CD10+/CD19+/CD20+/µchain+
CD34-/CD10+/CD19+/CD20+/µchain-

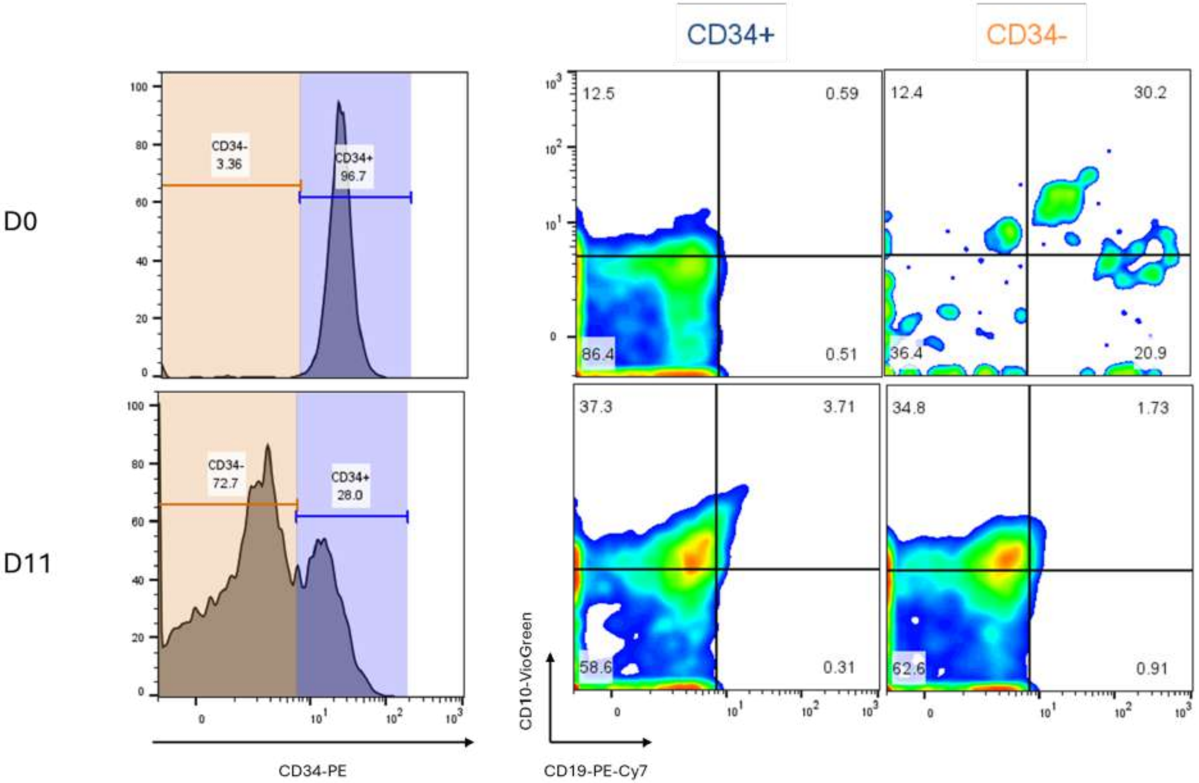
CD34+
CD34-
D0
D11
CD34-
3.36
CD34+
96.7
CD34-
72.7
CD34+
28.0
CD34-PE
CD10-VioGreen
CD19-PE-Cy7

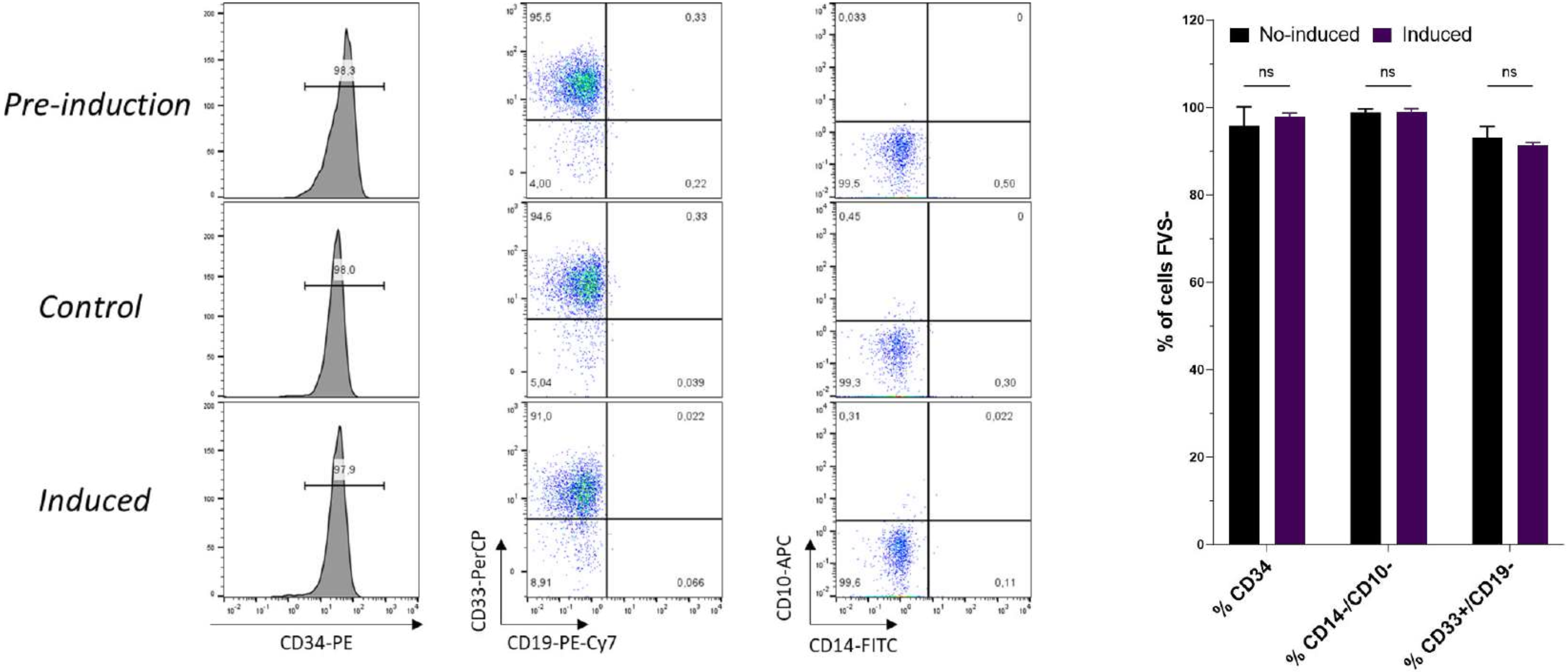

Pre-induction
Control
Induced
98,3
98,0
97,9
CD34-PE
95,5
0,33
4,00
0,22
94,6
0,33
5,04
0,039
91,0
0,022
8,91
0,066
CD33-PerCP
CD19-PE-Cy7
0,033
0
99,5
0,50
0,45
0
99,3
0,30
0,31
0,022
99,6
0,11
CD10-APC
CD14-FITC
No-induced
Induced
ns
ns
ns
% of cells FVS-
120
100
80
60
40
20
0
% CD34
% CD14-/CD10-
% CD33+/CD19-

Antibody	Clone	Supplier
FVS		BD Biosciences
CD14-PB450	M5E2	BD Biosciences
CD3-PB450	UCHT1	BD Biosciences
CD20-FITC	2H7	Beckman Coulter
CD34-PE	8G12	BD Biosciences
CD19-PEC7	J3-119	Beckman Coulter
CD10-APC	ALB-1	Beckman Coulter
µchain-APC-H7	MHM-88	BD Biosciences
KappaLC-APCVio770	IS11-24D5	Miltenyi Biotec
CD179a-APC	HSL96	Miltenyi Biotec
CD43-PerCP	DF-T1	Miltenyi Biotec
CD33-PerCP	D3HL60.251	Beckman Coulter
CD14-FITC	M5E2	BD Biosciences
CD90-FITC	5E10	BD Biosciences
CD34-PE	581	BD Biosciences
CD73-PECy7	AD2	BD Biosciences
CD105-APC	266	BD Biosciences
CD45-APCH7	2D1	BD Biosciences
CD166-PB450	3A6	BD Biosciences
CD146-FITC	P1H12	BD Biosciences
CD106-PE	51-10C9	BD Biosciences
CD39-APCH7	TU66	BD Biosciences

Primer	Sens/antisens	Size	%GC	TM	Sequence
Pax-5	Sens	20	60	59	CCG-CCA-GAG-GAT-AGT-GGA-AC
	Antisens	20	55	58,2	GAT-GGA-ACT-GAC-GCT-AGG-CA
IKZF1	Sens	20	55	58,2	ATG-GGG-AAG-AAT-GTG-CGG-AG
	Antisens	20	55	58,3	CCG-GAA-TGC-AGC-TTG-ATG-TG
IKZF3	Sens	20	55	58,9	AAG-AAG-TGC-GGA-GGC-AAG-AC
	Antisens	20	55	58,4	GCA-GTG-GTC-ACA-CCG-ATA-CA
PU-1	Sens	21	52,4	58,4	CTA-TAC-CAA-CGC-CAA-ACG-CAC
	Antisens	21	52,4	58,4	GTG-AAG-TTG-TTC-TCG-GCG-AAG
EBF-1	Sens	21	57,1	58,1	CGG-AAG-GTA-CGC-CCT-CTT-ATC
	Antisens	20	60	58,6	CGT-CGT-CCA-TCC-TTC-ACT-CG
E2A	Sens	20	55	57,4	TCT-ACT-CCC-CGG-ATC-ACT-CA
	Antisens	20	60	59,1	CGT-CGT-AGC-TGG-GCG-ATA-AG

Primer	Sens/antisens	Size	%GC	TM	Sequence
GAPDH	Sens	20	50	57,9	AAG-GTG-AAG-GTC-GGA-GTC-AA
	Antisens	20	50	58,2	CTT-GAC-GGT-GCC-ATG-GAA-TT
CD19	Sens	20	55	58,3	AGC-CTA-GAG-CTG-AAG-GAC-GA
	Antisens	20	50	58,4	AAT-GCC-CAC-AAG-GGA-ACA-CA
IRF4	Sens	20	60	59,3	GGG-CAA-GCA-GGA-CTA-CAA-CC
	Antisens	20	55	58,8	CTC-TTG-TTC-AAA-GCG-CAC-CG
IRF8	Sens	20	55	58,6	GGA-TCC-CTT-GGA-AAC-ACG-CT
	Antisens	20	55	58,2	CAA-AGC-ACA-GCG-TAA-CCT-CG
RAG-1	Sens	20	50	58,9	GTG-AGG-GAA-ATG-AGT-CTG-GT
	Antisens	20	55	59,2	CCT-AAT-GGG-TCC-CCT-AAG-CT
RAG-2	Sens	19	52,6	57,3	GCC-ATG-ATC-TAC-TGC-TCT-C
	Antisens	19	52,6	58,9	CTT-TGG-GGA-GTG-TGT-AGA-G
TdT	Sens				
	Antisens				
AID	Sens	23	47,8	58,6	CAC-TGG-ACT-TTG-GTT-ATC-TTC-GC
	Antisens	21	47,6	58,4	CGT-AAG-TCAA-CCT-CAT-ACA-GG

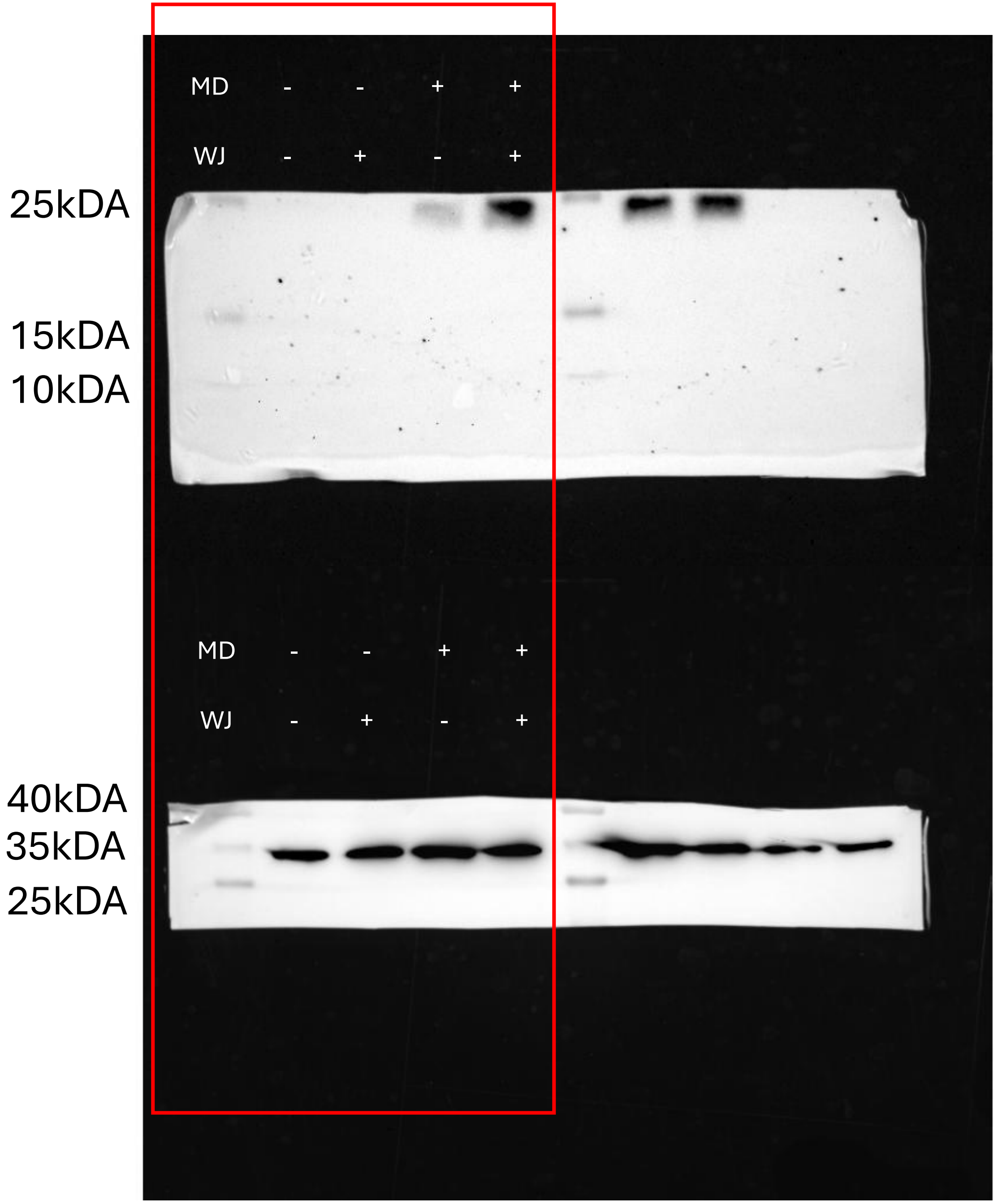

PageRuler molecular weight (Thermofisher)

Light chain Kappa = Size 22,5kDA

GAPDH = size 36kDA

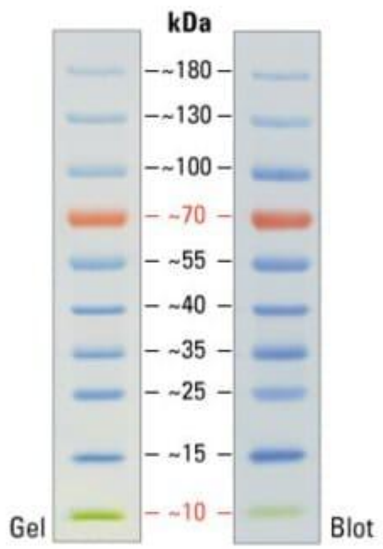

Figure 1. Western blot analysis of LCK expression and GAPDH loading control.

Representative immunoblots showing LCK (25 kDa) expression (upper panel) and GAPDH (37 kDa) used as loading control (lower panel) in the indicated samples. Equal amounts of total protein were loaded per lane. Membranes were incubated with the indicated primary antibodies followed by appropriate HRP-conjugated secondary antibodies and revealed by chemiluminescence.

In red, the figure cropped in our manuscript
